# What is the optimal timing for implant placement in oral cancer patients? A scoping literature review

**DOI:** 10.1111/odi.13312

**Published:** 2020-03-19

**Authors:** Jamie M. Alberga, Nathalie Vosselman, Anke Korfage, Konstantina Delli, Max J. H. Witjes, Gerry M. Raghoebar, Arjan Vissink

**Affiliations:** ^1^ Department of Oral and Maxillofacial Surgery University Medical Center Groningen University of Groningen Groningen The Netherlands

**Keywords:** ablative surgery, dental implants, dental prosthesis, head and neck cancer, primary placement, timing

## Abstract

**Background:**

Oral cancer patients can benefit from dental implant placement. Traditionally, implants are placed after completing oncologic treatment (secondary implant placement). Implant placement during ablative surgery (primary placement) in oral cancer patients seems beneficial in terms of early start of oral rehabilitation and limiting additional surgical interventions. Guidelines on the ideal timing of implant placement in oral cancer patients are missing.

**Objective:**

To perform a scoping literature review on studies examining the timing of dental implant placement in oral cancer patients and propose a clinical practice recommendations guideline.

**Methods:**

A literature search for studies dealing with primary and/or secondary implant placement in MEDLINE was conducted (last search December 27, 2019). The primary outcome was 5‐year implant survival.

**Results:**

Sixteen out of 808 studies were considered eligible. Both primary and secondary implant placement showed acceptable overall implant survival ratios with a higher pooled 5‐year implant survival rate for primary implant placement 92.8% (95% CI: 87.1%–98.5%) than secondary placed implants (86.4%, 95% CI: 77.0%–95.8%). Primary implant placement is accompanied by earlier prosthetic rehabilitation after tumor surgery.

**Conclusion:**

Patients with oral cancer greatly benefit from, preferably primary placed, dental implants in their prosthetic rehabilitation. The combination of tumor surgery with implant placement in native mandibular bone should be provided as standard care.

## 1 INTRODUCTION

**Figure 1:**
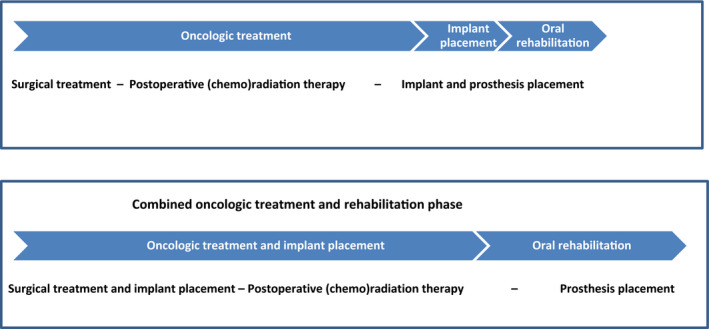
Timing of oncologic treatment and oral rehabilitation

The general treatment timeline for oral cancer patients consists of diagnostics, surgical treatment followed by postoperative (chemo)radiation therapy depending on the surgical margins and specific tumor properties, or solely (chemo)radiation therapy. Traditionally, oral rehabilitation comes last, that is, after the oncologic treatment when the oral mucosa is completely healed (Figure 1). Oral function after treatment for a malignancy in the oral cavity is often compromised due to changed anatomy after surgery and/or the oral sequelae of radiotherapy like xerostomia and trismus (de Groot et al., 2019; Kamstra et al., 2011). Sometimes, teeth need to be extracted during ablative surgery because of their location in proximity to the tumor or as part of a preradiation screening examination (Spijkervet, Schuurhuis, Stokman, Witjes, & Vissink, 2020). This compromised oral condition also leads to a decrease in oral function and possible a negative effect on nutritional status and quality of life (Jager‐Wittenaar et al., 2011). Fabrication of functional prostheses, frames, and conventional partial dentures is often difficult to achieve after oncologic treatment and in some cases even impossible (Curtis & Cantor, 1974; Petrovic, Rosen, Matros, Huryn, & Shah, 2018).

Dental implants have shown to be a great asset in oral cancer patients and provide good results (Said et al., 2017; Schoen et al., 2007). When dental rehabilitation based on implants first was introduced in oral cancer patients, they were often placed after oncologic treatment (secondary implant placement) (Kim & Ghali, 2011). This implies an additional surgery, for irradiated patients under antibiotic prophylaxis, and an additional treatment burden in older patients with often multiple comorbidities. When pretreatment hyperbaric oxygen treatment is advised, the treatment burden increases even more (Spijkervet, Brennan, Peterson, Witjes, & Vissink, 2019). When offering implant treatment in a secondary phase, patients are less likely to accept or undergo additional procedures, even when they could benefit from an implant‐supported prosthesis (Flores‐Ruiz et al., 2018; Schoen et al., 2007).

Implants can also be placed during tumor surgery (primary implant placement) (Schoen, Reintsema, Raghoebar, Vissink, & Roodenburg, 2004). An advantage of this treatment sequence is that most of the osseointegration takes place during the recovery phase, saving the burden of additional surgery and a considerable amount of time. The patient can function with an implant‐supported prosthesis much earlier after completion of oncologic treatment (Petrovic et al., 2018). Disadvantages are possibly improper placement of implants due to the changed anatomy during surgery or the risk of implants not being used because of tumor recurrence or patients passing away before a prosthesis can be made (loss of resources). The effects of radiotherapy on the osseointegration process and implant survival rates are also subject of debate (Chrcanovic, Albrektsson, & Wennerberg, 2016), and primary implant placement is not always available in the hospital setting (Shugaa‐Addin, Al‐Shamiri, Al‐Maweri, & Tarakji, 2016; Tanaka, Chan, Tindle, MacEachern, & Oh, 2013).

Guidelines when to ideally start oral rehabilitation with dental implants in oral cancer patients are lacking. Several systematic reviews have been published, mainly dealing with timing of secondary implant placement after radiotherapy (Claudy et al., 2013; Filho, Souza, & Santos, 2015; Granström, 2003; Nooh, 2013; Schiegnitz, Al‐Nawas, Kämmerer, & Grötz, 2014). Claudy et al. (2013) reported that dental implant placement between 6 and 12 months after radiotherapy was associated with a 34% higher risk of failure and therefore suggest waiting periods over 1 year after radiotherapy. On the contrary, it has been suggested that implant placement just becomes more critical over time because of the ongoing progressive decrease in healing capacity of bone after radiotherapy (Granström, 2003; Granström, Bergström, Tjellström, & Brånemark, 1994). Other studies showed no significant relationship between time interval and dental implant survival rates (Nooh, 2013; Filho et al., 2015). The implant survival rate in patients with a history of radiotherapy seems to be more associated with the location of the implants (more implant loss in the maxilla than in the mandible) than with the time after radiotherapy (Buddula et al., 2012). Far less studies on primary implant placement have been published. A systematic review by Barber, Butterworth, and Rogers (2011) on primary implant placement provides an extensive literature overview, but no clear conclusions or recommendations were made. The latter systematic review also included case reports and studies on patients with benign lesions, which could have influenced the outcome. The authors of another systematic review highlighted the importance of timing of implant placement and concluded that they could not extract scientific evidence for the optimal timing of implant placement (Shugaa‐Addin et al., 2016).

Before being able to propose guidelines for optimal timing of implant placement in head and neck cancer patients needing radiotherapy, the following questions have to be answered: (a) what is the optimal timing of dental implant placement in oral cancer patients with regard to implant survival and functional outcomes, and (b) can all oral cancer patients benefit from primary placement or is this method of treatment only suitable for specific patient groups. As implant treatment and techniques have evolved during the last decade, we comprehensively reviewed the literature on the timing of implant placement in oral cancer patients to compose recommendations for clinical practice with regard to optimal timing of implant placement in this category of patients.

## 2 METHODS

A search was conducted in MEDLINE (from 1995 through October 16, 2019) on October 16, 2019, according to the syntax rules of the database. Key words and their combinations were used to identify relevant studies (Table S1). The titles and abstracts from all the searches were reviewed.

**Figure 2:**
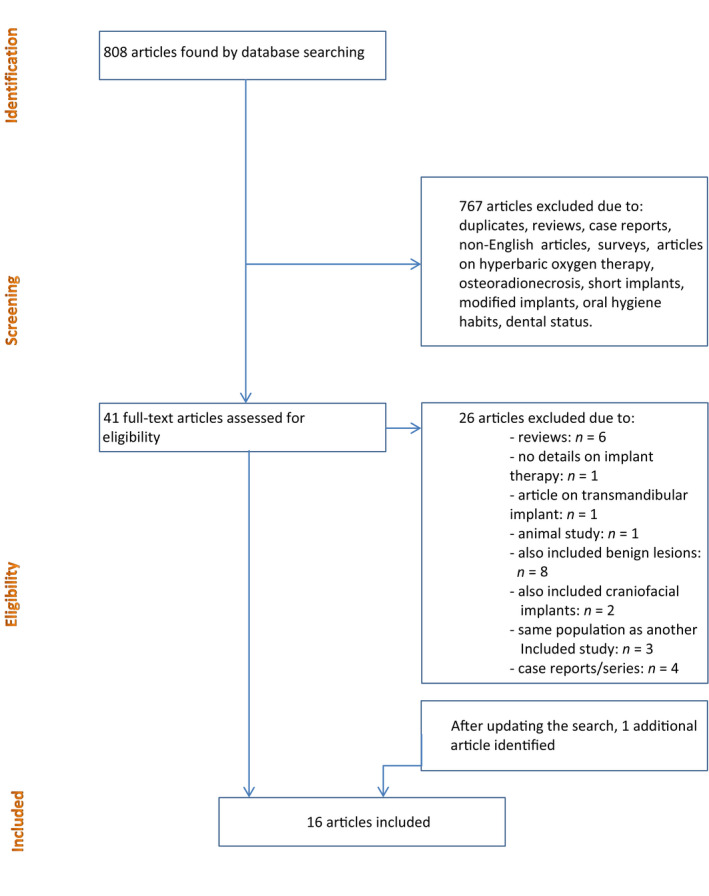
Flowchart of study selection procedure

Inclusion criteria were studies published in English regarding primary or secondary implant placement in oral cancer patients, cohort studies, case–control studies, (randomized) controlled trials. Review articles, animal studies, case reports, case series with <10 patients, and studies regarding extra‐oral craniofacial implants were excluded. When it was not clear from the title and abstract if the paper dealt with implant placement in the upcoming irradiated (primary implant placement) or already irradiated (secondary implant placement) mandible or maxilla, the full text was reviewed and the article was included or excluded. Forty‐one full‐text articles were assessed followed by exclusion of 26 articles due to various reasons (Figure 2). Furthermore, hand searches of the references of retrieved articles were carried out. The search was updated on December 27, 2019, and one additional article was included. Eventually, 16 studies were included.

### 2.1 Data extraction

The following data were collected from the studies: patient demographics (age, oncologic diagnosis, patients’ dental status before treatment), type of oncological treatment, timing of endosseous or zygomatic implant placement (primary, secondary), implant system, site of implant placement, type of tissue implants were inserted into (native or augmented bone), time until loading, implant loss, implant survival ratios, complications, perioperative measurements, type of prosthesis, and follow‐up period (Tables 1, 2, 3). When available, the time span between (implant) surgery and prosthesis placement, and the time between radiotherapy and secondary implant placement were recorded.

**Table 1:** General characteristics of eligible studies

	First author	Year	Study type	*N*	Patient age (mean, range)	Oncologic diagnosis	Patients' dental status	Site of implant placement	Implant system	Tissue implants inserted to	RT	Radiation dose in region of implant	Timing of implant placement
1	Flores‐Ruiz	2018	Retrospective	17	30–60	Epidermoid carcinoma, osteosarcoma, lymphoepithelioma	Edentulous and partially edentulous	Mandible and maxilla	Unknown	Native and grafted bone	Yes (47%)	Not reported	Secondary
2	Curi	2018	Retrospective cohort study	35	46–94	SCC	Not reported	Mandible and maxilla	Replace Select Tapered; Nobel Biocare	Native bone	>50 Gy	>50 Gy	Secondary
3	Rana	2016	Retrospective	46	60	Oral cancer	Not reported	Mandible and maxilla	Biomet 3i	Native bone	Yes	Not reported	Secondary
4	Wu	2016	Retrospective	34	52.1	SCC, ACC, mucoepidermoid carcinoma, malignant ameloblastoma, nasopharynx tumor, acinic cell carcinoma	Not reported	Mandible and maxilla	Straumann, Nobel Biocare	Native and grafted bone (4 ilium bone, 18 fibula grafts)	Yes <50 Gy	Not reported	Secondary
5	Sammartino	2011	Prospective	77	55.8, 28–63	Head and neck cancer	Edentulous and partially edentulous	Mandible and maxilla	Solid screw with microstructured surface	Native bone	Yes all	Not reported	Secondary
6	Nelson	2007	Retrospective	93	59, 26–89	Malignant intraoral tumor	Edentulous and partially edentulous	Mandible and maxilla	CAMLOG, Steri‐Oss (Nobel Biocare), Straumann	Native and grafted bone (ilium and fibula bone)	Yes (29/93) patients with up to 72 Gy)	Not reported	Secondary
7	Yerit	2006	Retrospective	71	57.8, 16–84.1	Oral cancer (majority SCC T2‐T4)	Not reported	Mandible	IMZ (Friadent), Frialit II (Friadent), Xive (Friadent)	Native and grafted bone (iliac bone)	Up to 50 Gy	Not reported	Secondary
8	Visch	2002	Prospective	130	62, 34–87	Head and neck cancer	Not reported	Mandible and maxilla	Hydroxyapatite‐coated titanium. Dyna, Screw‐Vent Implants	Native bone	Yes (50−72 Gy)	Not reported	Secondary
9	Seikaly	2019	Prospective	30	57	Malignant disease not further specified	Not reported	Mandible and maxilla	Not reported	Grafted bone (fibula free flap)	7/15 primary; 9/15 secondary	Not reported	Primary and secondary
10	Butterworth	2019	Prospective	49	70, 13–92	SCC, ACC, sarcoma, adenocarcinoma, melanoma, rhabdomyosarcoma, ameloblastoma, pleomorphic adenoma, ORN	Edentulous and dentate	Upper jaw/ zygoma	Not reported	Native bone	Yes 16/49	Not reported	Primary and secondary (2 groups)
11	Wetzels	2017	Retrospective cohort study	97 (79 prim. 18 s)	66.25 (prim.), 68.32 (sec.)	SCC, merkel cell carcinoma, salivary gland carcinoma	Edentulous	Mandible and maxilla	Branemark Nobel Biocare (primary), Astra/Straumann (secondary)	Native bone (both primary and secondary	55% (prim.), 53% (sec.)	Not reported	Primary and secondary (2 groups)
12	Ch'ng	2016	Retrospective	246	59.0	ACC, adenocarcinoma, ameloblastic carcinoma, desmoid tumor, fibrosarcoma, melanoma, osteosarcoma, SCC, hemangioendothelioma	Unknown	Mandible and maxilla	Astra Tech	Native and grafted bone (67 fibula free flaps)	165/246 (60−72 Gy)	Not reported	Primary and secondary
13	Wetzels	2016	Prospective	56	67–70	Intraoral malignancies not further specified	Edentulous	Mandible	Branemark (primary), Astra + Straumann (secondary)	Native and grafted bone. Primary: 2 free vascularized bone flaps. Secondary: 4 free vascularized bone flaps	Yes	Not reported	Primary and secondary
14	Mizbah	2013	Retrospective	99	Not reported	Primary SCC	Edentulous	Mandible	Branemark (primary), Frialit (delayed)	Native bone	Primary 47/99. Secondary 17/29	Not reported	Primary and secondary
15	Korfage	2014	Prospective cohort	164	64.8, 39–88	SCC	Edentulous	Mandible	Branemark (Nobel Biocare)	Native bone	Yes (64)	Not reported	Primary
16	Schepers	2006	Retrospective	48	64.8 (men), 68.1 (women)	Primary SCC in oral cavity	Edentulous	Mandible	Branemark	Native bone	Yes (21/48)	10−68 Gy	Primary

Note

Studies number 1–8: Studies on secondary implant placement. Studies number 9–14: Studies on both primary and secondary placed implants. Studies number 15– 16: Studies on primary implant placement.

Abbreviations: ACC, adenoid cystic carcinoma; FFF, fibula free flaps; Gy, Gray; ORN, osteoradionecrosis; prim, primary; RT, radiotherapy; SCC, squamous cell carcinoma; sec, secondary.

**Table 2:** Data on implant treatments and implant survival of included studies

First author	Primary implant placement (*N*)	Secondary implant placement (*N*)	Total no. of implants	Time after RT until implant placement	Time until loading	Number of implants per patient	Implant loss	Implant survival rate	Follow‐up period
Flores‐Ruiz	0	17	106 (15 implants in grafted bone; 43 in the maxilla)	70% >2 yrs after radiotherapy	Not reported	Not reported	13 failed (9 maxilla, 4 mandible; 9 native bone, 4 grafted bone)	90.1% native bone. 73.3% grafted bone. 79.2% maxilla. 87.7% mandible. Overall 87.7%	5 yrs
Curi	0	0	169 (79 implants in the maxilla, 90 implants in the mandible)	1–92 mo	6 mo	Not reported	12 implants (3 during healing period and 9 lost after loading)	92.9% 5 yrs	7.43 yrs
Rana	0	46	162 (70 implants in the maxilla)	6–24 mo	Not reported	Not reported	52	65% maxilla 71% mandible	5 yrs
Wu	0	34	187 (63 implants in maxilla; 68 implants in native bone)	6–12 mo	0.8 yrs	Not reported	27	93.2% native bone. 93.8% grafted bone. 87.3% maxilla. 97.5% mandible Overall 93.6%	5 yrs
Sammartino	0	77	188 (42 implants in the maxilla, 146 in the mandible)	At least 6 mo. Mean time: 9.4 mo	6 mo (mandible), 8 mo (maxilla)	2 mandible; 3–5 maxilla	2 implants lost in mandible; 18 implants lost in maxilla	98.4% in mandible; 57.1% in maxilla. 90.5% in <12 mo after RT. 82.2% in >12 mo after RT	3 yrs
Nelson	0	93	435 (281 implants in the maxilla; 95 implants in grafted bone)	Minimum 6 mo	3 mo mandible, 6 mo maxilla	3–8	43 implants	Maxilla 70% after 4 yrs. Overall implant survival 92%, 84%, and 69% after 3.5, 8.5, and 13 yrs. Implant survival rates for implants in grafted bone unknown	13 yrs
Yerit	0	71	316 (171 in iliac bone)	1.41 yrs after surgery	>6 mo	Not reported	44 implants	Overall: 95%, 94%, 91%, and 75% after 2, 3, 5, and 8 yrs Irradiated: 93%, 90%, 84%, and 72% after 2, 3, 5, and 8 yrs. Grafted bone: 96%, 96%, 96%, and 54% after 2, 3, 5, and 8 yrs	5.4 yrs
Visch	0	130	446 (108 implants in the maxilla, 338 implants in the mandible)	6 mo to 22 yrs	6 mo	Not reported	64 implants	Overall: 78% 10 yrs Maxilla 60%, mandible 85%. 10 yrs	10 yrs
Seikaly	15	15	110 (57 implants primary; 53 implants secondary). Number of implants in maxilla/ mandible not reported	Not reported	6 mo	Not reported	2 implants lost in both groups	Overall: 96%	1 yr
Butterworth	27 patients and 75 zygoma implants + 14 standard	22 patients and 56 implants + 16 standard	131 zygomatic implants. Additionally 30 dental implants in the maxilla	Not reported	Primary 1.7 mo, secondary 9.3 mo	NA	9 zygoma implants	12 mo estimated 94%, 60 mo estimated 92%	2–110 mo
Wetzels (2017)	79 patients and 207 implants. 52 implants never loaded	18 patients and 43 implants placed 528 days after surgery	268 (in primary group 18 additional implants were placed postsurgery)	At least 6 mo disease‐free	3 mo (non‐irradiated), 6 mo (irradiated)	2–4	17 primary implants failed (6.7%), 12 mandible, 5 maxilla. 5/17 due to implant‐related cause. Secondary group 3 implants lost (7%) due to loss of flap in which implants were placed. In primary group, 32% implants failed due to patient death, versus 7% in secondary group due to patient death	Higher cumulative implant survival rates in secondary group. Primary 60%. Secondary 86%	5 yrs
Ch'ng	115 during ablative surgery. 41 primary RT	90	1,132 (243 implants in fibula free flaps; 618 implants in native mandible, 271 in native maxilla)	Not reported	Not reported	2–9 in fibula free flap	Overall 42/1132 lost	Mandible 97.4%. Maxilla 95.3%. Fibula free flap 92.6% Overall 96.3% at follow‐up. 5 yrs 94.9%	5 yrs
Wetzels (2016)	18 patients and 40 implants	9 patients and 19 implants placed 568 days after surgery	59	Unknown (secondary implants were placed at least 1 yr after ablative surgery)	Not reported	2 or 3	In primary group, 3/40 implants lost. In secondary group, 3/19 implants lost	Primary 92.5%. Secondary 84.2%	5 yrs
Mizbah	99	29	163	At least 1 yr no recurrence	3 mo (non‐irradiated), 6 mo (irradiated)	2–4	24 (primary) = 9.6%, 6 (secondary) = 9.2%	Primary 90.4%; secondary 90.8%	5 yrs
Korfage	164	0	524	–	3 mo (non‐irradiated), 9 mo (irradiated)	2–4	31 (irradiated patients), 5 (non‐irradiated patients)	93.1%	Up to 14 yrs
Schepers	48	0	139	–	9 mo (irradiated), 4.7 mo (non‐irradiated)	2–4	2/61 (irradiated), 0/78 (non‐irradiated)	96.7% (irradiated), 100% non‐irradiated	29.6 mo

Abbreviations: mo, months; RT, radiotherapy; yrs, years.

**Table 3:** Data on type of prosthetic rehabilitation, functional outcomes, and perioperative measurements

First author	Reported clinical measurements	Peri‐implant bone loss	Type of prosthesis	Functional outcomes	Prophylaxis	Complications	Overall conclusion
Flores‐Ruiz	None	Not reported	Overdenture, fixed prostheses	None	None	Not reported	There is no consensus as to the time needed to achieve successful survival after placement of implants
Curi	None	Not reported	Overdentures	Patient satisfaction, mastication, speech, aesthetics	Clindamycin 4 × 300 mg 1 week starting 1 day before treatment; HBO (37.1%)	Not reported	Dental implants in head and neck cancer patients with RT are a viable treatment alternative with a high degree of satisfaction. The type of RT may require special consideration. IMRT has less implant failure than conformal RT
Rana	Not reported	Not reported	Cemented and removable overdentures	None	None	Not reported	Further research is required in this field to improve aesthetics and quality of life
Wu	BI,GI,PI	1.2 ± 0.4 to 1.6 ± 0.6 mm	Fixed and removable dentures	None	HBO (14 patients)	65 prosthetic maintenance procedure (abutment/screw loosening). No surgical complications reported	Dental implants are more successful in the mandible than in the maxilla. No difference in survival rates between patients who received HBO and who did not. The restoration of oral function in radiotherapy patients with tumor resection using implant‐supported prostheses is a viable treatment option
Sammartino	None	Panoramic and periapical	Overdentures, maxillary obturators	None	No HBO	Not reported	Implant therapy can be considered in irradiated patients when from an oncologic standpoint the tumor prognosis is benign and the risk of recurrence is poor. Higher implant success rates in the mandible and in irradiated implant sites with a dosage no more than 40−50 Gy
Nelson	None	Not reported	Fixed and removable dentures	None	Irradiated patients clindamycin 300 mg 1 day preoperatively and 3 days postoperatively	Technical complications: Replacement of 11 bar‐retained dentures. 2 patients with mucosa ulcers after loss of retention of the removable denture. 3 patients with dehiscence and disturbed wound healing	The mean 10.3‐year survival rate was low, and there was no statistically significant difference in implant survival between irradiated and non‐irradiated patients. The increased failure rate was caused by the higher mortality rate of the patients; it was not the result of lack of osseointegration. There was no difference between implant survival in grafted and non‐grafted patients
Yerit	None	Not reported	Removable denture	None	No HBO	1 patient with a pathological fracture of the mandible leading to loss of 3 implants	Shorter implant survival in irradiated and grafted bone. No difference in survival between implant placed < or >12 months after RT. Surgical and prosthetic implant rehabilitation of tumor patients offer long‐term results with favorable implant survival rates
Visch	None	Not reported	Not reported	None	AB prophylaxis. No HBO	Not reported	After a postirradiation interval of six months, the influence of time on implant survival is not significant. Bone‐resection surgery in the jaw where the implant is placed has a significantly negative influence on implant survival. Implant location is the most dominant variable influencing implant survival (more implant loss in maxilla than in the mandible)
Seikaly	None	Not reported	Not reported	Not reported	HBO	Primary placement: 2 major complications (hematoma, pulmonary embolism) and 7 minor complications (tachycardia, atelectasis, wound infection/breakdown, partial fibular skin graft loss). Secondary placement: 2 major complications (flap venous congestion and pneumonia) and 5 minor complications (wound infection/breakdown)	Primary implant placement in fibula free flaps reduced the duration of time to complete treatment from 6.1 to 1.8 years. The reduction in treatment time was not associated with a statistically significant increase in complications
Butterworth	None	Not reported	Oral (fixed and removable) and facial prostheses	QOL. No significant problems with swallowing	NA	No significant complications in primary implant group. Secondary implant group: 2 patients with an infection of the skin overlying the zygomatic body. 2 patients with peri‐implant bone loss. Small number of patients with screw loosening and screw fracture	Primary implant placement should be the gold standard. Access for zygomatic implant placement is much improved at primary resective surgery. There is a trend toward worse survival rates in secondary placement
Wetzels et al. (2017)	None	Not reported	Overdenture	None	6 patients HBO in secondary group	Primary implant group: 52 implants were never loaded. 5 patients with ORN. Secondary implant group: 5 patients with ORN	More functional overdentures in primary group. Prosthetic rehabilitation 484 days earlier in primary implants. Timing of placement does not affect viability of implants.
Ch'ng	None	Not reported	Removable denture	None	Not reported		More implant losses in fibula free flaps. RT adversely affects implant survival in FFF but not in the native mandible or maxilla. The sequence of RT in relation to implant placement did not significantly affect the implant survival rate, except in fibula free flaps. Irradiation might be considered a relative contraindication to implant placement in osseous free flaps. No conclusion on timing
Wetzels et al. (2016)	None	Not reported	Overdenture	Bite force, masticatory performance	HBO in irradiated patients in secondary group	1 patient with ORN (not adjacent to the still functional implants)	There is a strong indication of superior bite force and masticatory performance after 5 years in primary group when compared to postponed placement. It seems that primary placement is superior to secondary placement
Mizbah	None	Not reported	Overdenture	None	HBO in irradiated patients in secondary group	Not reported	Using primary placement, more patients benefit and receive their overdentures at an earlier stages (20 months earlier) compared to secondary placement
Korfage	Periodontal indices	Panoramic	Overdenture	EORTC QLQ, OHIP	HBO in 3 patients who developed ORN	5 patients with ORN in proximity to the implants. Pathological mandible fracture in 1 patient with a recurrent tumor and ORN	More limitations in oral function and less satisfaction in irradiated patients. Better oral function with than without prosthesis. A large number of patients with oral cancer in whom implants are inserted during resection may benefit at an early stage from an overdenture and develop good function, satisfaction. Primary insertion should be routinely incorporated into surgical planning. More implant loss in irradiated patients
Schepers	None	Not reported	Removable denture	None	Not reported	No patients developed ORN. No other complications reported	Success of prosthetic rehabilitation on implants inserted during ablative surgery is independent of whether postoperative RT is applied. Primary implant placement in edentulous mandibles appears to have advantages over secondary implant placement in patients with oral SCC

Abbreviations: AB, antibiotic; ACC, adenoid cystic carcinoma; BI, bleeding index; EORTC QLQ, European Organization for Research and Treatment of Cancer Quality of Life Questionnaire; FFF, fibula free flaps; GI, gingiva index; Gy, Gray; HBO, hyperbaric oxygen; IMRT, intensity‐modulated radiation therapy; OHIP, Oral Health Impact Profile; ORN, osteoradionecrosis; PI, plaque index; PORT, postoperative radiotherapy; RT, radiotherapy; SCC, squamous cell carcinoma.

### 2.2 Statistical analysis

Quantitative data‐synthesis was performed for the studies reporting 5‐year dental implant survival rates of primary placed implants and secondary placed implants. Studies which did not report on the 5‐year implant survival rate were not included in the quantitative analysis. The pooled 5‐year implant survival rates were analyzed using a random effects model. Analyses were performed with Comprehensive Meta‐Analysis software, Version 3 (CMA; Biostat).

## 3 RESULTS

Sixteen out of 808 papers were considered eligible for our study, and one additional article was included after updating the search (Figure 2). These 16 studies provided data on a total of 4,449 implants, of which 753 implants were placed in grafted bone (osseous free flaps). The majority of studies (68.8%) had a retrospective design. Preoperative dental status (edentulous or dentate) was not always reported. Patients received an implant‐supported removable or fixed prosthesis. A variety of malignancies in the head and neck region was reported. Oncologic treatment consisted of tumor surgery in addition to radiotherapy. Three articles reported on including patients who were treated with chemotherapy (Ch’ng et al., 2015; Flores‐Ruiz et al., 2018; Yerit et al., 2006). Eight articles reported solely on secondary implant placement (Curi, Condezo, Ribeiro, & Cardoso, 2018; Flores‐Ruiz et al., 2018; Nelson, Heberer, & Glatzer, 2007; Rana et al., 2016; Sammartino, Marenzi, Cioffi, Tete, & Mortellaro, 2011; Visch, van Waas, Schmitz, & Levendag, 2002; Wu, Huang, Zhang, Zhang, & Zou, 2016; Yerit et al., 2006), two studies described patients with only primary placed implants (Korfage et al., 2014; Schepers, Slagter, Kaanders, Hoogen, & Merkx, 2006), and six articles described both primary and secondary implant placement (Butterworth, 2019; Ch’ng et al., 2015; Mizbah et al., 2013; Seikaly et al., 2019; Wetzels et al., 2016; Wetzels, Meijer, Koole, Merkx, & Speksnijder, 2017). In all studies, implants were placed in a 2‐stage manner. When mentioned, the number of implants per patient ranged between 2 and 4 in the interforaminal region of the mandible (Korfage et al., 2014; Mizbah et al., 2013; Schepers et al., 2006; Wetzels et al., 2016). Only one study reported the number of implants placed in the maxilla (3–5) (Sammartino et al., 2011). From the available data, a total of 987 implants were placed in the maxilla and 131 zygomatic implants were placed in the zygomatic bone.

### 3.1 Implant survival

**Figure 3:**
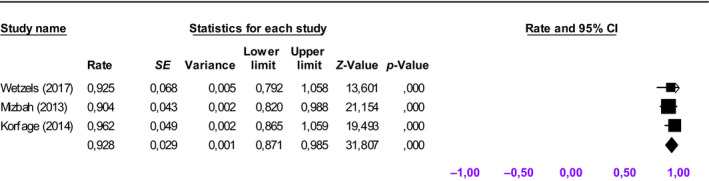
Forest plot for cumulative weighted 5‐year implant survival rate for primary implant placement

**Figure 4:**
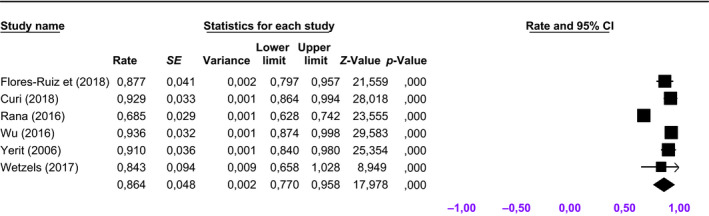
Forest plot for cumulative weighted 5‐year implant survival rate for secondary implant placement

The pooled 5‐year survival rate for primary placed implants was 92.8% (95% CI: 87.1%–98.5%) (Figure 3), while the pooled implant survival rate for secondary placed implants was 86.4% (95% CI: 77.0%–95.8%) (Figure 4). The 5‐year implant survival rate of primary placed implants tended to be higher compared to secondary placed implants. Survival ratios for dental implants placed in vascularized bone grafts varied between 54% and 93.8% (Table 2). The implants in vascularized bone grafts were placed in a secondary procedure. Implant survival ratios in native maxillary bone ranged between 57.1% and 95.3%. One study focused mainly on zygomatic implants (Butterworth, 2019) and reported a 5‐year implant survival rate of 92%.

### 3.2 Time between ablative surgery, implant placement, radiotherapy, and prosthesis placement

In two studies on primary implant placement, a healing period of 6 months after radiotherapy was applied before second‐stage surgery (Korfage et al., 2014; Seikaly et al., 2019). In another study, a waiting period of 9 months was applied (Schepers et al., 2006). Time from tumor surgery and implant placement until prosthesis placement from three studies varied from 6.3 to 21.4 months (Korfage et al., 2014; Mizbah et al., 2013; Seikaly et al., 2019).

In the secondary setting, there was a preference for waiting at least six months after completing radiotherapy before starting implant treatment. Some studies even preferred to wait at least 1 year (Mizbah et al., 2013; Wetzels et al., 2016). Generally, patients had to wait more than 1 year after oncologic treatment before the oral rehabilitation was started. In the article by Flores‐Ruiz et al. (2018), 70% of the patients started with implant therapy even later than 2 years after oncologic therapy. The study of Seikaly et al. (2019) reported a mean time to prosthetic rehabilitation of 73.1 months. For zygomatic implants, there was also a difference between primary and secondary placed implants (median time until loading 1.7 months vs. 9.3 months) (Butterworth, 2019).

### 3.3 Functional outcomes

Korfage et al. (2014) described that irradiated patients experience more limitations in oral function than those who were not. Chewing ability decreased over time in irradiated patients, but there was still a better oral function in patients with a prosthesis than in patients without a prosthesis (Korfage et al., 2014). A more objective method for measuring oral function was applied in the study by Wetzels et al. (2016) by determining masticatory performance. The authors showed an increased masticatory performance in all patients with implant‐supported prostheses, supporting the assumption that implants are beneficial for improved oral function in oral cancer patients.

### 3.4 Complications

Intra‐ and postoperative complications of dental implant placement were uncommon. The most common reported complication was osteoradionecrosis (ORN) in irradiated patients (Ch’ng et al., 2015; Korfage et al., 2014; Wetzels et al., 2016, 2017). The ORN rate varied between 1.8% and 7.7%. One study reported a pathologic fracture (Ch'ng et al.), but it was unclear whether the fracture occurred because of implant placement. In the study with zygomatic implants, infection of the overlying skin in secondary placed implants occurred in two patients (Butterworth, 2019). There were no complications in the group with primary placed zygomatic implants. Other complications like wound infections, wound breakdown, and partial fibular skin graft loss were described for implants placed in fibula free flaps (Seikaly et al., 2019). Technical complications in primary and secondary placed implants included incorrect implant positioning. In the study of Korfage et al. (2014), six out of 164 patients (3.7%) with primary placed implants did not receive an implant‐supported prosthesis due to incorrect implant positioning. Another study reported 17.7% unused implants after primary placement (17.7%) (Mizbah et al., 2013) due to incorrect positioned implants and tumor‐related factors.

## 4 DISCUSSION

Timing of dental implant placement in oral cancer patients is a subject of continuing debate. Although most of the studies that were considered to be eligible for the review had retrospective study designs and studied implant placement in heterogeneous patient populations, it can be concluded that dental implant placement, irrespective of the timing of implant placement, is a reliable treatment option for head and neck cancer patients. Both primary and secondary implant placement show an acceptable overall implant survival. Comparison between both groups showed a tendency for a higher 5‐year implant survival rate in primary implant placement. This trend, however, did not reach statistical significance. Implants placed in the maxilla tended to have lower survival ratios than implants placed in the mandible. The lower implant survival ratios in maxillary bone might be related to the thinner cortical bone of the maxilla. For zygomatic implants however, 5‐year implant survival rates of 92% were reported (Butterworth, 2019). An explanation for these favorable outcomes could be that zygomatic implants are inserted in highly cortical bone of the zygoma, leading to a high initial stability. Because of their length, these implants may also be situated outside of the radiated field, therefore avoiding toxic radiation dosages. At this moment, functional results for zygomatic implants seem good and complication rates low, but guidelines on the optimal workflow are not yet available (Hackett, El‐Wazani, & Butterworth, 2020).

A great advantage of primary implant placement is the earlier prosthetic rehabilitation after tumor surgery. The latter is a great asset, also because it is not uncommon that head and neck cancer patients refuse the burden of undergoing the secondary implant placement, notwithstanding the great advantage they could experience from an implant‐supported oral rehabilitation (Schoen et al., 2007).

The costs and potential “loss of resources” from implants not being used are an important issue in primary implant placement. The percentage of incorrect placed implants varied between the studies. We believe that with the help of 3D technology, implant positioning (especially in difficult cases) can be further improved as has already been demonstrated in small groups for primary implant placement (Chuka et al., 2017). Placing implants during ablative surgery slightly lengthens the operating time, but the extra costs and burden to the patient of an additional secondary implant procedure under local anesthesia are prevented.

As stated earlier, precision of implant placement can be improved further with 3D technologies or surgical design and simulation (SDS). In both primary and secondary implant placement, 3D planning software can be used to assess the amount of available bone height and width for dental implants after resection and to assess the ideal location for the implants from a prosthetic point of view (Witjes, Schepers, & Kraeima, 2018). The use of SDS has resulted in a high percentage of implant utilization (96%) for mandibular defects constructed with fibula free flaps (Seikaly et al., 2019). We therefore consider the availability of 3D planning techniques a necessity in the reconstruction of oral cancer patients with complex (continuity) defects.

Only one study on primary implant placement in osseous free flaps for larger defects was considered eligible for our review (Seikaly et al., 2019). In this prospectively conducted study, dental implants were placed in bone grafts (mainly fibula grafts) during the ablative procedure. This resulted in a significant reduction of time to rehabilitation and percentage of patients rehabilitated. Most reports on implant placement in osseous free flaps include heterogeneous patient populations and show successful treatment outcomes with implant survival ratios between 80% to 100% (Kumar et al., 2016; Sozzi, Novelli, Silva, Connely, & Tartaglia, 2017). Jackson, Price, Arce, and Moore (2016) compared primary to secondary implant placement in fibula free flaps and found no difference in implant survival between primary and secondary implantation, and between non‐irradiated and irradiated patients (Jackson et al., 2016). The 1‐year results of Sandoval et al. (2019) in 10 patients with primary placed implants in fibula free flaps show that the presence of dental implants in fibula free flaps does not lead to more postoperative complications or an increase of radiotherapy‐related toxicities. Despite these promising results, correct placement of dental implants in osseous free flaps during ablative surgery is technically challenging as reviewed by Bodard, Salino, Bemer, Lucas, and Breton (2011). One way of partially reducing these challenges is through the use of occlusion‐driven reconstructions aided by 3D planning, as is demonstrated in the article of Seikaly et al. (2019). However, the essential difference in tissues covering the grafted bone of the fibula and native mandibular bone remains. The presence of subcutaneous tissue and the absence of keratinized gingiva could affect implant survival and peri‐implant health. The patients should be strictly monitored to see whether complications might occur on the long run. Additional thinning or correction of the overlying skin paddle is sometimes necessary during second‐stage surgery (Kumar et al., 2016; Patel, Kim, & Ghali, 2019). Regarding functional outcomes, Wijbenga, Schepers, Werker, Witjes, and Dijkstra (2016) concluded from their systematic review that despite high implant survival ratios, it is not possible to state what the effect of implant‐supported dental prostheses is after reconstruction with a fibula free flap, again mainly due to the diversity of methods used to assess functional outcomes. Awad et al. (2019), however, concluded in their systematic review that 61% of patients with a vascularized fibula flap receiving dental rehabilitation reported good oral function and was able to consume a normal diet. The latter authors, however, did not make a statement on the timing of implant placement in vascularized fibula flaps. With respect to timing of implant placement in osseous free flaps, it is generally advised to insert implants primarily only in patients with benign lesions (Chang et al., 1997; Patel et al., 2019). In our clinic, we prefer to place dental implants as much as possible in the remaining native mandibular bone (during ablative surgery) in order not to jeopardize the vitality of the vascularized fibula flap. As mechanical stability comes from the more anterior region of the mandible, this approach is successful in lateral and antero‐lateral defects.

**Figure 5:**
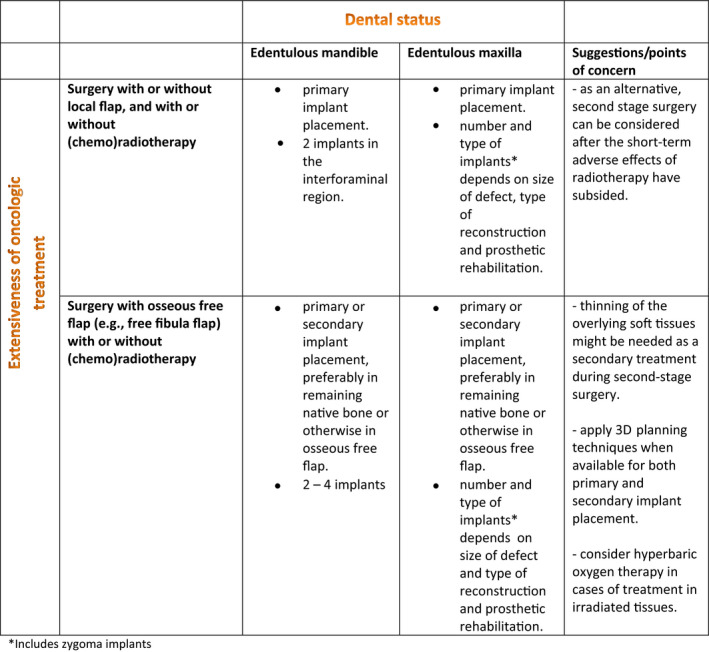
Recommendations for dental implant placement to support implant‐retained overdentures in head and neck cancer patients. *Includes zygoma implants

Limitations of this scoping review include, as stated earlier, the retrospective study designs, heterogeneous patient populations, exclusion of non‐English papers, the use of one database, and the fact that screening by carried out by assessor. These factors could result in bias. Due to the unavailability of large prospective studies on the timing of implant placement in oral cancer patients, the treatment of choice will mainly depend on surgeon experience and preference. However, based on the findings in the current study and our own experience in treating these patients, we composed treatment recommendations on the timing of implant placement in patients with malignant intraoral tumors (Figure 5). We realize that these recommendations may not be applicable to all hospital settings as 3D planning software and the financial resources for primary implant placement may not be available in every center.

## 5 CONCLUSION

Based on the studies included in this review, as far as the timing of implant placement is regarded, we propose to routinely combine tumor surgery with implant placement in native mandibular bone as standard care (primary implant placement). The functional benefits of primary implant placement outweigh the risk of leaving (some) implants unused. For more complex reconstructive cases, a personalized treatment approach (aided by 3D technologies) is necessary and is more often in need of a secondary implant placement. It seems that primary placement of zygomatic implants is accompanied by a high implant survival and good oral rehabilitation although more research is needed on this particular topic.

## CONFLICT OF INTEREST

The authors have stated explicitly that there are no conflicts of interest in connection with this article.

## AUTHOR CONTRIBUTIONS

J.A. conducted the literature search, analyzed the data, and wrote the initial manuscript. J.A. and K.D. performed the statistical analysis. K.D. designed the figures (forest plots). A.K., N.V., and M.W. contributed to the analysis of the results. All authors discussed the results and contributed to the final manuscript at all stages.

## Supporting information

Table S1
