# Supplementary material for: What is the optimal timing for implant placement in oral cancer patients? A scoping literature review
Source: Oral Dis. 2020 Mar 19;27(1):94–110. doi: 10.1111/odi.13312 (PMC7818452; doi:10.1111/odi.13312)
Supplement: Supplementary file 1 — Table S1 [file ODI-27-94-s001.docx]

*Supplementary table 1. Search strategy.*

| **Database** | **Search Terms** |
| --- | --- |
| Medline | *("Head and Neck Neoplasms"[Mesh] OR Head and Neck Neoplasm*[tiab] OR Head and Neck cancer*[tiab] OR cancer of head and neck[tiab] OR head and neck oncol*[tiab] OR Head and Neck malignan*[tiab] OR head and neck tum*[tiab] OR Upper Aerodigestive Tract Neoplasm*[tiab] OR mouth neoplasm*[tiab] OR oral cancer*[tiab] OR oral neoplasm*[tiab] OR oropharynx malignan*[tiab] OR oropharynx tum*[tiab]) AND ("Dental Implants"[Mesh] OR "Dental Implantation, Endosseous"[Mesh] OR "Dental Prosthesis, Implant-Supported"[Mesh] OR implant*[tiab] OR denture*[tiab]) AND (Primary placement*[tiab] OR primary insert*[tiab] OR ablation surg*[tiab] OR ablative surg*[tiab] OR "Time"[Mesh] OR time*[tiab] OR timing[tiab] OR delay*[tiab] OR sequence*[tiab])* |
